# Global impact of accelerated plant breeding: Evidence from a meta-analysis on rice breeding

**DOI:** 10.1371/journal.pone.0199016

**Published:** 2018-06-14

**Authors:** Bert Lenaerts, Yann de Mey, Matty Demont

**Affiliations:** 1 Centre for Environmental Sciences, UHasselt, Hasselt, Belgium; 2 Agri-food Policy Platform, International Rice Research Institute (IRRI), Los Baños, Philippines; 3 Department of Earth and Environmental Sciences, KU Leuven, Leuven, Belgium; 4 Business Economics Group, Wageningen University & Research, Wageningen, the Netherlands; Texas Tech University, UNITED STATES

## Abstract

Rice breeders in Asia and elsewhere in the world have long overlooked trying to shorten the time it takes to develop new varieties. Plant breeders have proposed a technique called Rapid Generation Advance (RGA) as a way to accelerate the results of public rice breeding programs. However, little is known about RGA’s potential impact. Here, we present the first results of a global impact study of RGA. More specifically, we calculated the multiplicator effects of RGA on the research benefits generated by conventional rice breeding programs and applied them to a meta-analysis of selected impact studies in the literature. These insights are a first crucial step in developing a targeted approach for disseminating RGA technology among rice breeders to accelerate the impact of their public rice breeding programs around the world. We show that the additional benefits due to time savings are considerable and offer some insights into the economics of breeding. Our results confirm that the adoption of accelerated breeding would lead to substantial advantages to rice breeding programs and the earlier variety release leads to significant economic benefits to society. This can be important to policy makers when reshaping their public breeding methods and optimising their return on research investments in breeding.

## Introduction

Rice farmers experienced great transformation during the Green Revolution. Much of this can be attributed to plant breeding [1] as exemplified by the introduction of the semidwarf variety IR8 in 1966. Since the 1970s, plant breeding—or genetic improvement—has been the major success story of research and development in agriculture, especially in the developing world. Using rice as an example, production more than doubled, from 257 million tons in 1966 to 600 million tons in 2000 [2]. By 2000–2001, 65% of rice-cropped land was covered by modern varieties in Bangladesh [3]. During 1998–2000, modern variety adoption reached 81% in India [4]. By 2002, the adoption rate was 94% for modern varieties in Vietnam [5]. The Philippines and Indonesia also experienced high levels of adoption (70–90%). In contrast, the Green Revolution was much less successful in Thailand and Nepal in terms of modern variety adoption [6].

Improved rice varieties were—and remain—the main driving force for further agricultural development and are the foundation to providing a reliable rice supply for about 3.5 billion people worldwide. As breeding has played a crucial role in the past, future challenges place an even higher demand on enhanced breeding processes. These challenges include climate change, depleting natural resources, and a rising population of which a significant part already faces hunger today. So, improved varieties need to be developed and released faster. With today’s tighter budgets, more cost-efficient breeding operations are needed.

Astonishingly, the irrigated breeding program at the International Rice Research Institute (IRRI)—the largest nonprofit agricultural research centre focusing on rice breeding in Asia and Africa—has remained largely unchanged for decades. Lenaerts et al. [7] show that the conventional pedigree method is still very widespread across national and international breeding institutes. In light of the challenges outlined above, awareness is growing about the need to update current breeding methods. Currently, IRRI’s breeders are re-organising their overall breeding operation through what is called Transforming Rice Breeding (TRB), a Bill & Melinda Gates Foundation-funded project that will make primarily irrigated breeding pipelines more efficient and targeted [8]. TRB’s main policy objective is demonstrating how the transformation of IRRI’s breeding activities can serve as a model for the similar transformation of many other public breeding institutions, particularly in developing countries. The general focus lies on transforming public-sector breeding programs into more efficient and effective pipelines that are market-driven and product-oriented comparable to those in the highly competitive private-sector programs for commercial crops.

A key change considered in the TRB project includes shortening the breeding process by using Rapid Generation Advance (RGA) which is also known as single seed descent (SSD) [9]. This method shortens the time required to fix breeding lines (i.e. make them genetically homozygous) and also the breeding cycle (i.e. time from “cross to cross”). Ultimately, this also reduces the variety development time. Doubled haploids and molecular markers are other measures being used to reduce breeding time. However, as these methods are technically more complex and their operations more expensive, this study focuses on RGA. In essence, RGA’s line fixation method is an alternative to the commonly used pedigree technique. Traditionally, the two processes of breeding are selection for traits and inbreeding for homozygosity. With RGA, only the inbreeding is done with no selection so plants do not have to be grown in the field under real-life conditions, which saves much time and resources [9].

However, a constraint to developing a sound public breeding policy is the lack of a strong base of evidence regarding RGA’s benefits and impact. First, an economic impact assessment must be undertaken to make sure that RGA is indeed worthwhile from an economic perspective. Second, this base of evidence must be large enough and understandable to convince breeders to adopt RGA.

Unfortunately, studies focusing on the economic benefits of accelerating the rice breeding process are remarkably scarce. Breeding operations tend to take several years and, as a consequence, data collection can be tedious. Furthermore, models developed to estimate the benefits from the release of varieties emanating from RGA require a lengthy set of parameters to be calibrated. These parameters are highly region-specific and so are the corresponding benefit models. Since impact studies on accelerated breeding processes require these benefit models to be run twice—pre- and post-implementation of the RGA-based breeding program—these studies are scarce. Brennan [10] and Brennan and Martin [11] provide some evidence of shortening the breeding process for wheat, although the impact of shorter breeding times is not the main focus of their research. Brennan [10] found an increase in benefits of US$ 190,000 for southern New South Wales (Australia) after a one-year time reduction while Brennan and Martin [11] estimate the returns to be worth almost US$ 14 million for northwestern Mexico following a two-year reduction. Pandey and Rajatasereekul [12] provide a helpful reference for calculating the impact of shortening the breeding process in rice. They estimate the benefit at US$ 39 million for Northeast Thailand after a two-year reduction. All of the aforementioned studies use a discount rate of 5% and assume the breeding method enables a reduction in time that does not raise breeding costs substantially. Alpuerto et al. [13] also provide an interesting example of economic benefits from reducing the breeding process in rice using molecular methods. According to them, incremental economic benefits coming from an earlier release of salinity-tolerant and phosphorus deficiency-tolerant rice in Bangladesh, India, Indonesia, and the Philippines are between US$ 50 million and US$ 900 million over a period of 25 years (5% discount rate).

The contributions presented in this article are fourfold. First, we present a novel and particularly parsimonious approach to deal with the difficulties of traditional impact studies of accelerated breeding processes highlighted above. In essence, we mathematically derive an exact multiplicator, which can be used to calculate incremental benefits from earlier benefit release. We calculate these multiplicator effects for single projects as well as for larger breeding programs consisting of multiple breeding projects. Second, this multiplicator can be applied to existing impact studies of breeding techniques since it depends only on two parameters, i.e., the discount rates used and the number of years saved. Therefore, we conduct a meta-analysis of recent rice breeding impact studies and greatly expand the current evidence base of impact assessment on accelerated breeding processes. Third, we complement these calculations with a sensitivity analysis to gain some insight into the benefit structure of discounted incremental benefits. Apart from insights offered by Pandey and Rajatasereekul [12], to the best of our knowledge, this extensive approach has not been taken before. Fourth, we discuss policy implications for public and private breeding institutions.

## Analytical framework

In this section, we develop our theoretical framework to calculate the incremental public benefits from varietal improvement research that can be attributed to RGA. Hereafter, benefits from varietal improvement research attributed to a conventional breeding method will be referred to as “research benefits” and the difference in benefits between a conventional breeding method and RGA as “incremental benefits”, unless specified otherwise. Note that these are gross benefits, not net benefits. We always refer to discounted benefits, unless specified otherwise. Table 1 provides a list of all symbols used throughout this section.

**Table 1:** List of symbols used.

Symbol	Definition
*B*_CM_, *B*_RGA_	Research benefits for pedigree (conventional method) and RGA, respectively
*b*	Conventional breeding process (in years)
*m*	Lag between the availability of improved varieties and farmers’ adoption (in years)
*l*	Lifespan of the variety used (in years)
*i*	Discount rate used
*B*_*t*_	Annual undiscounted benefit from variety release in year *t*
*r*	Reduction in the breeding process (in years)
*β*_RGA_	Relative difference in benefits of RGA compared to pedigree
*n*	Number of breeding projects in a breeding program after the inception project
*K*	Measure of the scale of the breeding project
*a*	Relative lateness of the benefits throughout the lifespan of the variety

### Breeding projects

**Fig 1:**
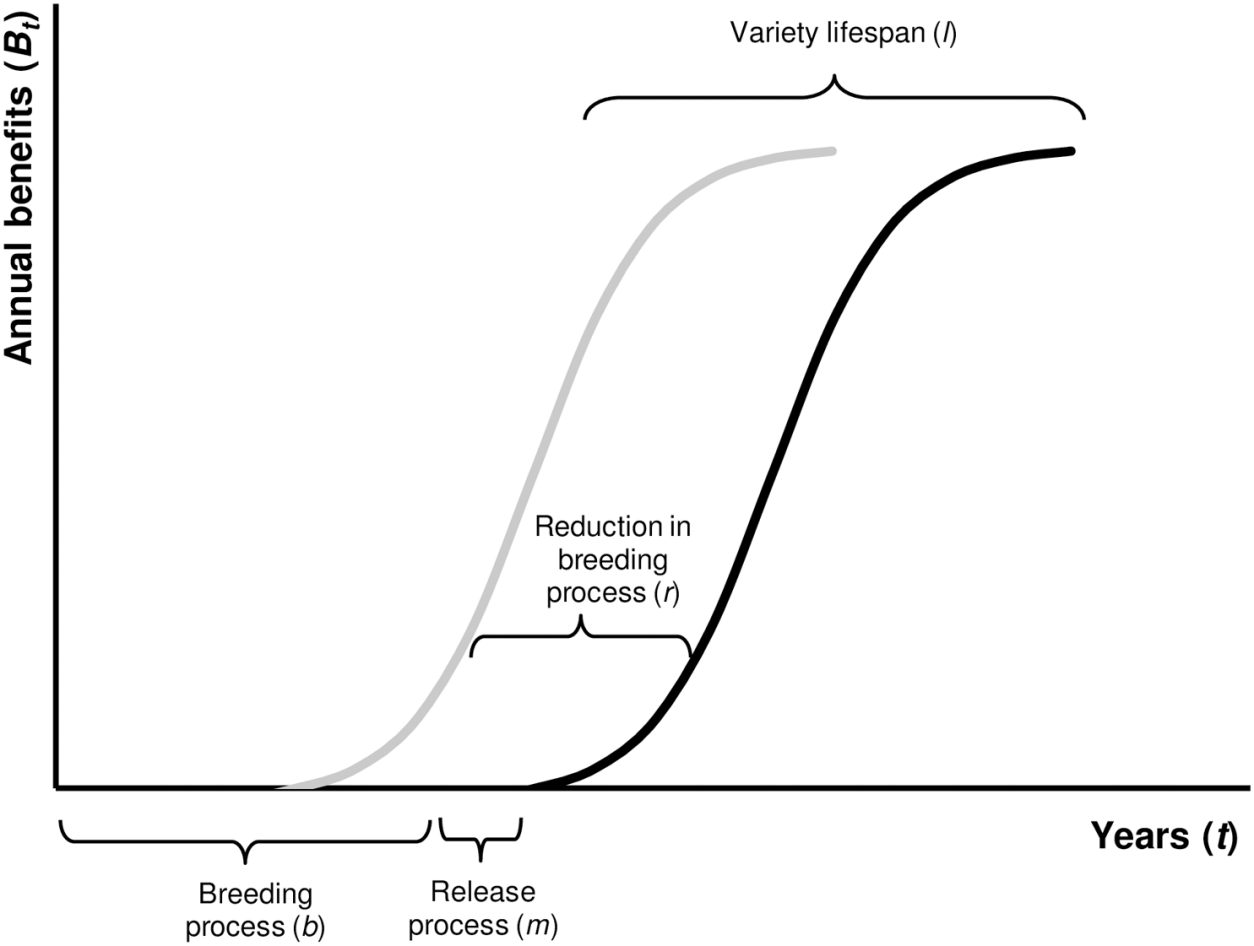
Time dimension of a breeding project for the conventional (pedigree, black line) and RGA (grey line) breeding method.

A fundamental feature of plant breeding is that costs of breeding are incurred early on while (undiscounted) benefits from adoption of improved varieties arrive only late in the breeding project. Fig 1 visualises the timing of a breeding project involving (i) the breeding process (*b*), (ii) the release process (*m*), and (iii) the variety’s lifespan (*l*). The method we present relies on the crucial assumption that research benefits are the same for the RGA breeding method and the conventional breeding method, i.e., the lifespan of the variety remains the same and annual undiscounted benefits are comparable. The only difference in benefits arises from an earlier release and hence an earlier adoption of the new variety. This assumption seems reasonable as RGA only speeds up the breeding process and is not focused specifically on generating varieties that achieve yield or quality-related gains in the short run that exceed those of a regular breeding project. Note that it is not necessary to assume annual undiscounted benefits are constant.

This principal assumption provides a useful starting ground for comparing breeding processes and the benefits that arise from them. To derive the relative incremental benefits, we start off with standard discounting formulas, both for the conventional and RGA breeding methods. Research benefits from the variety’s lifespan for the conventional method for one single breeding project (*B*_CM_) are calculated as follows:

$$B_{CM}=\sum_{t=b+m+1}^{b+m+l}\frac{B_{t}}{{\left(1+i\right)}^{t}},$$ (1)

where *B*_*t*_ is the annual undiscounted benefit from variety release for the conventional method in year *t*, *i* the discount rate used, *l* the lifespan of the variety used (in years), *b* the conventional breeding process (in years) and *m* the lag between the availability of improved varieties and farmers’ adoption (in years).

As the benefit streams for RGA are identical to those of the conventional method, but start and end sooner, the research benefits from the variety’s lifespan for the RGA method (*B*_RGA_) can be computed as follows:

$$B_{RGA}=\sum_{t=b+m+1-r}^{b+m+l-r}\frac{B_{t}^{'}}{{\left(1+i\right)}^{t}}=\sum_{t=b+m+1-r}^{b+m+l-r}\frac{B_{t+r}}{{\left(1+i\right)}^{t}}$$ (2)

where *r* is the reduction in the breeding process (in years) and $B_{t}^{'}$ is the annual undiscounted benefit from variety release for the RGA method in year *t*.

For the benefits from RGA, we substitute the summation index, *t + r = s* and consequently change the summation limits:

$$B_{RGA}=\sum_{s=b+m+1}^{b+m+l}\frac{B_{s}}{{\left(1+i\right)}^{s-r}}={\left(1+i\right)}^{r}\sum_{s=b+m+1}^{b+m+l}\frac{B_{s}}{{\left(1+i\right)}^{s}}.$$ (3)

Or when we rename the summation index *s* back to *t* (note that the act of consecutively substituting and renaming back a summation index is a common operation in summation algebra):

$${B_{RGA}=(1+i)}^{r}\sum_{t=b+m+1}^{b+m+l}\frac{B_{t}}{{\left(1+i\right)}^{t}}.$$ (4)

Next, the incremental benefits from using RGA relative to the research benefits from the conventional method (*β*_RGA_) can be calculated as follows:

$$\beta_{RGA}=\frac{B_{RGA}-B_{CM}}{B_{CM}}=\frac{B_{RGA}}{B_{CM}}-1=\frac{{\left(1+i\right)}^{r}\sum_{t=b+m+1}^{b+m+l}\frac{B_{t}}{{\left(1+i\right)}^{t}}}{\sum_{t=b+m+1}^{b+m+l}\frac{B_{t}}{{\left(1+i\right)}^{t}}}-1$$

$$\beta_{RGA}={\left(1+i\right)}^{r}-1.$$ (5)

When calculating this relative difference in benefits (*β*_RGA_), it is clear from the derivation of the discounting formulas used that the relative difference is independent of the length of the breeding process, the lifespan of the developed variety and the lag in adoption, and only depends on the discount rate (*i*) used and the reduction in breeding time (*r*). Table 2 shows an overview of relative incremental benefits from time savings (*β*_RGA_) by varying these two parameters. Since this method is also applicable for calculating the benefits from a reduction in adoption lag, e.g., due to improved extension programs, a wide range of time savings is reported. Note that this study assumes adoption lags to be the same for varieties produced by the conventional and RGA breeding method and only considers a reduction in breeding time. For the common case where the breeding process is reduced with two years (*r* = 2) [8–9], the formula can be approximated as follows:

$${\beta_{RGA}=(1+i)}^{2}-1={2i+i}^{2}\approx 2i$$ (6)

**Table 2:** Relative incremental benefits (*β*_RGA_) from time savings (%).

**Years saved**	**Discount rate (%)**									
	**1**	**2**	**3**	**4**	**5**	**6**	**7**	**8**	**9**	**10**
**0**	0	0	0	0	0	0	0	0	0	0
**1**	1	2	3	4	5	6	7	8	9	10
**2**	2	4	6	8	10	12	14	17	19	21
**3**	3	6	9	12	16	19	23	26	30	33
**4**	4	8	13	17	22	26	31	36	41	46
**5**	5	10	16	22	28	34	40	47	54	61
**6**	6	13	19	27	34	42	50	59	68	77
**7**	7	15	23	32	41	50	61	71	83	95

For discount rates up to 10%, the approximation error is less than 5%. For purposes of accuracy, however, the full formula will be used for the benefit analysis in this study.

When the benefits from the conventional breeding method (*B*_CM_) are taken as given, the incremental benefits from a single breeding project using RGA (Δ*B*_RGA_) can be presented as follows:

$${{\Delta B}_{RGA}=\beta_{RGA}∙B_{CM}=[(1+i)}^{r}-1]\;B_{CM}$$ (7)

### Breeding programs

By shortening a single breeding project (i.e., development of one variety), RGA has the potential to create more benefits in comparison with pedigree (conventional method). Most breeding institutes, however, continuously develop multiple varieties over time, adding up to a breeding program (i.e., series of breeding projects). Understandably, if multiple breeding projects are conducted, incremental benefits are even greater. However, as more breeding projects are added, each additional future project offers less net present value due to the process of discounting until eventually no additional discounted benefits can be gained. Although we argue that incremental benefits from a single breeding project are sufficient to make our case in favour of RGA, it might be interesting to know how program benefits approach their upper bound. Therefore, we expand our method to calculate the relative benefits from a number of repetitions of a single breeding project. Because discounted benefits eventually decline to zero, limits to infinity are also taken.

**Fig 2:**
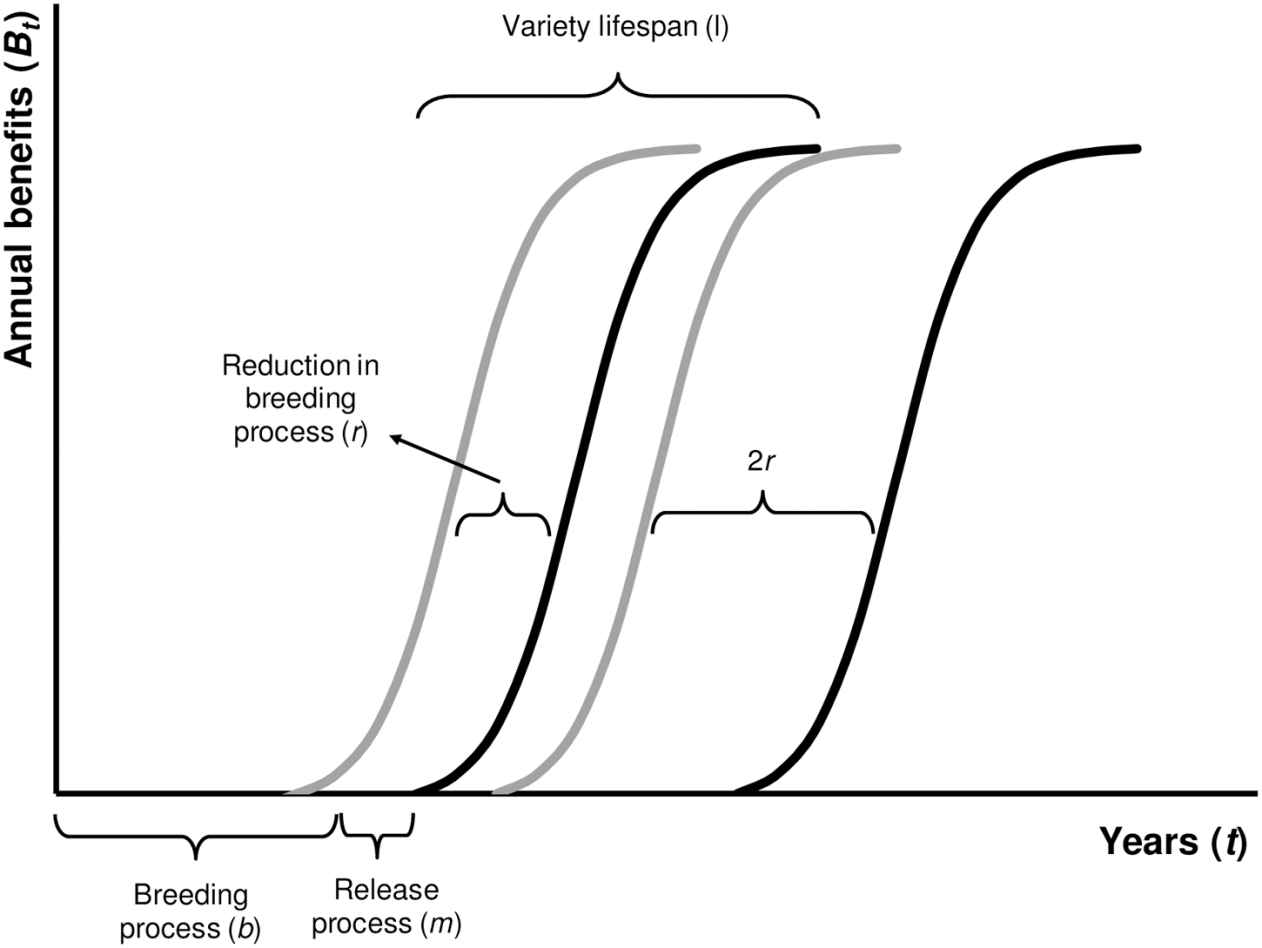
Time dimension of a breeding program with 2 breeding projects (*n* = 1) for the conventional (pedigree, black line) and RGA (grey line) breeding methods.

Total discounted benefits from a single breeding project using the pedigree method are still represented by *B*_CM_. The total lifespan (*l*) of the variety is captured completely in *B*_CM_. Immediately after the breeding period for a variety is completed and facilities such as land and greenhouses are released again, a new breeding project is started. Because a variety’s breeding process is smaller than its lifespan, the adoption pattern of new varieties overlaps partially with older ones (see Fig 2), and this effect is greater for RGA-bred varieties. As breeding is a value-generating process at the start of the agricultural supply chain, we assume that with more breeding (i.e., faster variety release), more value can be created. In this view, competition between older and newer varieties for market or land share is considered to be low. A first explanation for this is the presence of early and late adopters among famers. Rogers [14] defines the concept of innovativeness as “*the degree to which an individual or other unit of adoption is relatively earlier in adopting new ideas than the other members of a system*” (p. 22), and identifies several adopter categories: innovators, early adopters, early majority, late majority, and laggards. Going back to our assumption, we see that the adoption by farmers who adopt varieties lately is indeed not likely to be influenced by newly released varieties—which will in turn be adopted by early adopters. Alternatively, a new variety might cover a different market segment than the older one and consequently there is no interaction between the adoption rate of both varieties. Fig 2 shows the pattern of breeding for two breeding projects.

Introducing the parameter *n* that represents the number of breeding projects after the inception project and takes the value zero for the latter, benefits are first calculated for *n* + 1 breeding projects using pedigree. To do this, we repeat the inception breeding project *n* times with a delay of *b* years (length of breeding process for pedigree); we discount all projects and then sum these benefits. To calculate the delayed benefits from the *n* times repeated breeding project, we use the factor derived earlier in Eq (5). Because benefits are now delayed instead of brought forward, we divide by our factor instead of multiplying by it:

$$B_{CM,n}=\sum_{c=0}^{n}\frac{B_{CM}}{{\left(1+i\right)}^{bc}}=B_{CM}+\sum_{c=1}^{n}\frac{B_{CM}}{{\left(1+i\right)}^{bc}}.$$ (8)

We then use annuity formulas (adapted geometry series) to remove the summations:

$$\sum_{c=1}^{n}\frac{B_{CM}}{{\left(1+i\right)}^{bc}}=B_{CM}\frac{1-{\left(1+i\right)}^{-nb}}{{\left(1+i\right)}^{b}-1}$$ (9)

$$B_{CM,n}=B_{CM}+[B_{CM}\frac{1-{\left(1+i\right)}^{-nb}}{{\left(1+i\right)}^{b}-1}]$$

$$B_{CM,n}=B_{CM}[1+\frac{1-{\left(1+i\right)}^{-nb}}{{\left(1+i\right)}^{b}-1}]$$

$$B_{CM,n}=B_{CM}\frac{{\left(1+i\right)}^{b}-{\left(1+i\right)}^{-nb}}{{\left(1+i\right)}^{b}-1}.$$ (10)

Finally, we take the limit for *n* → ∞:

$$B_{CM,\infty }=B_{CM}\frac{{\left(1+i\right)}^{b}}{{\left(1+i\right)}^{b}-1}.$$ (11)

Next, benefits are calculated for *n* + 1 breeding projects using RGA. The calculation is analogous to the pedigree calculation with the difference of the original (inception) project starting *r* years earlier and also the repeated breeding projects thereafter only being delayed *b − r* years instead of *b* years:

$$B_{RGA,n}=\sum_{c=0}^{n}\frac{B_{RGA}}{{\left(1+i\right)}^{(b-r)c}}{=B}_{RGA}+\sum_{c=1}^{n}\frac{B_{RGA}}{{\left(1+i\right)}^{(b-r)c}}.$$ (12)

Again, we use annuity formulas (adapted geometry series) to remove the summations:

$$\sum_{c=1}^{n}\frac{B_{RGA}}{{\left(1+i\right)}^{(b-r)c}}=B_{RGA}\frac{1-{\left(1+i\right)}^{-n(b-r)}}{{\left(1+i\right)}^{\left(b-r\right)}-1}$$ (13)

$$B_{RGA,n}=B_{RGA}+[B_{RGA}\frac{1-{\left(1+i\right)}^{-n(b-r)}}{{\left(1+i\right)}^{\left(b-r\right)}-1}]$$

$$B_{RGA,n}=B_{RGA}[1+\frac{1-{\left(1+i\right)}^{-n(b-r)}}{{\left(1+i\right)}^{\left(b-r\right)}-1}]$$

$$B_{RGA,n}=B_{RGA}\frac{{\left(1+i\right)}^{b-r}-{\left(1+i\right)}^{-n(b-r)}}{{\left(1+i\right)}^{b-r}-1}.$$ (14)

We then substitute *B*_RGA_ using the earlier derived factor [Eq (5)]:

$$B_{RGA,n}={\left(1+i\right)}^{r}{∙B}_{CM}∙\frac{{\left(1+i\right)}^{b-r}-{\left(1+i\right)}^{-n(b-r)}}{{\left(1+i\right)}^{b-r}-1}.$$ (15)

Then, we take the limit for *n* → ∞:

$$B_{RGA,\infty }={{\left(1+i\right)}^{r}∙B}_{CM}∙\frac{{\left(1+i\right)}^{b-r}}{{\left(1+i\right)}^{b-r}-1}.$$ (16)

Finally, we calculate the relative increase in benefits from using RGA compared to using pedigree for a breeding program with *n* +1 breeding projects:

$$\beta_{RGA,n}=\frac{B_{RGA,n}-B_{CM,n}}{B_{CM,n}}=\frac{B_{RGA,n}}{B_{CM,n}}-1$$

$$\beta_{RGA,n}=\frac{\left[{{\left(1+i\right)}^{r}∙B}_{CM}∙\frac{{\left(1+i\right)}^{b-r}-{\left(1+i\right)}^{-n(b-r)}}{{\left(1+i\right)}^{b-r}-1}\right]}{B_{CM}∙\frac{{\left(1+i\right)}^{b}-{\left(1+i\right)}^{-nb}}{{\left(1+i\right)}^{b}-1}}-1$$

$$\beta_{RGA,n}=\frac{\frac{{\left(1+i\right)}^{b}-{\left(1+i\right)}^{-n(b-r)+r}}{{\left(1+i\right)}^{b-r}-1}}{\frac{{\left(1+i\right)}^{b}-{\left(1+i\right)}^{-nb}}{{\left(1+i\right)}^{b}-1}}-1.$$ (17)

**Fig 3:**
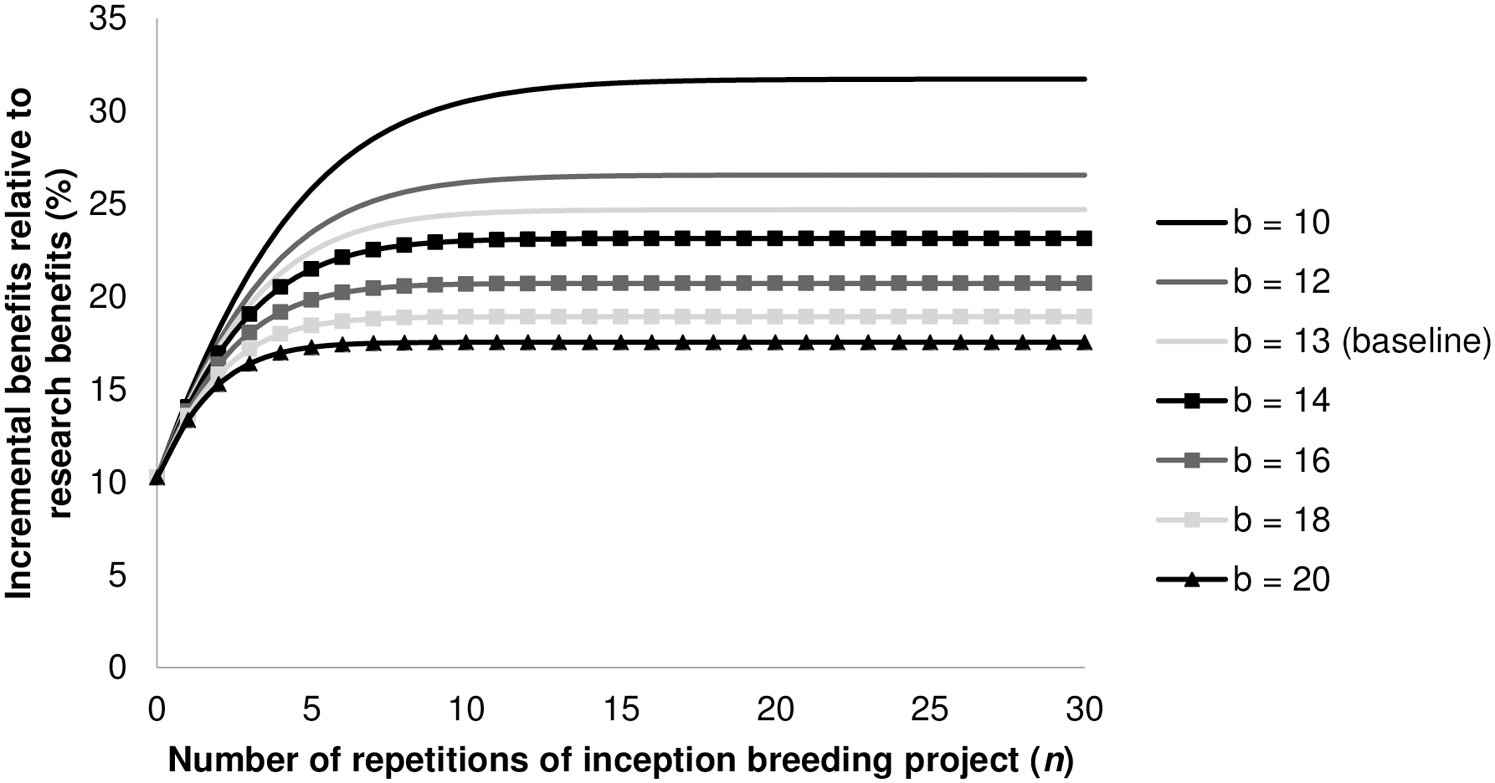
Relative incremental benefits for breeding programs (*β*_RGA,n_) with varying length of variety development time (*b*) based on Eq (17). *Note*: *r* = 2 and *i* = 0.05.

**Fig 4:**
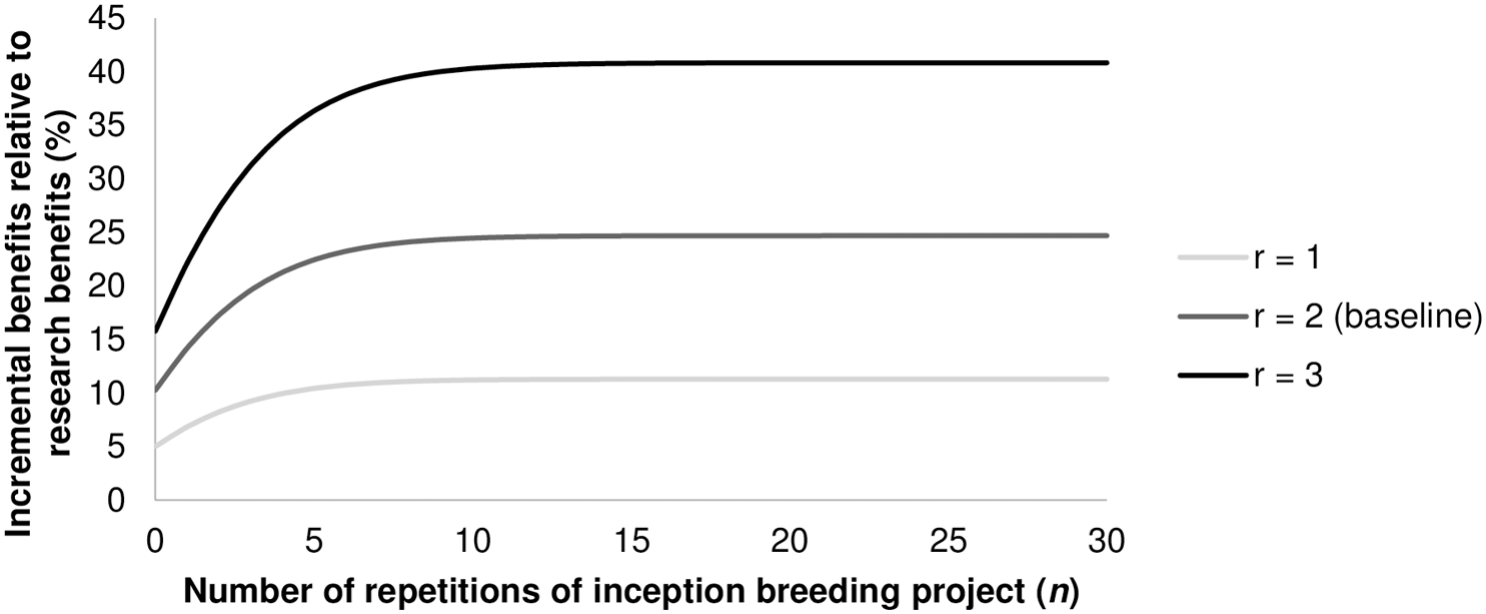
Relative incremental benefits for breeding programs (*β*_RGA,n_) with varying time savings (*r*) based on Eq (17). *Note*: *i* = 0.05 and *b* = 13.

For *n* = 0, Eq (17) collapses back to Eq (5). To illustrate the relative increase in benefits (*β*_RGA,*n*_), we take some values for the parameters from the literature on rice breeding. The length of breeding process (*b*) differs from country to country, but generally it takes at least 9 to 10 years for completion. However, it is several years longer in countries that only have one season per year [15]. Pandey and Rajatasereekul [12] report a breeding process of 13 years using conventional breeding methods. As before, we take the typical case in which the breeding process is reduced by two years [8–9]. We therefore plot Eq (17) starting from the baseline scenario with *r* = 2, *i* = 0.05 and *b* = 13. In Fig 3, we plot how the relative increase in benefits (*β*_RGA,*n*_) behaves as the breeding process varies from 10 up to 20 years, *ceteris paribus*. In Fig 4, time savings (*r*) are varied between one and three years since these are the possible reductions from using RGA [8–9].

As can be seen, after a certain number of repetitions, the benefits from using RGA converge to an asymptotic value; after this point, the incremental value of an additional breeding project is negligible. For the baseline scenario with *r* = 2, *i* = 0.05 and *b* = 13, benefits are already more than two times as high (23%) as a single breeding project (10%) when six breeding projects are conducted in series (Fig 3). From that point onwards, relative incremental benefits slowly start to converge towards 25%. For longer breeding processes, the maximum (limit) is reached much sooner. So breeding programs with longer breeding processes not only generate fewer benefits over time; they also benefit from RGA for a smaller amount of breeding projects. Nonetheless, even for a very long breeding process of 20 years, incremental benefits for the entire breeding program converge to a value more than one and a half times as large as for a single breeding project (10%). In short, we can say that, even for a small number of breeding projects with relatively long breeding processes, benefits are one and a half times that of a single breeding project for a two-year reduction at a 5% discount rate. For programs with small breeding processes and/or more breeding projects, benefits are even bigger by a factor between 2.5 and 3 compared to the single-project scenario. When we set the number of years saved from 2 to 1 for our baseline scenario, incremental benefits reduce by slightly more than 50%; a three-year reduction increases benefits by 65% compared to the two-year scenario (Fig 4).

Finally, we also take the limit for *n* → ∞ in Eq (17):

$$\beta_{RGA,\infty }=\frac{\frac{{\left(1+i\right)}^{b}}{{\left(1+i\right)}^{b-r}-1}}{\frac{{\left(1+i\right)}^{b}}{{\left(1+i\right)}^{b}-1}}-1=\frac{{\left(1+i\right)}^{b}-1}{{\left(1+i\right)}^{b-r}-1}-1.$$ (18)

The incremental benefits from a finite and infinite number of breeding projects using RGA as compared to using a conventional breeding method (Δ*B*_RGA,*n*_ and Δ*B*_RGA,*∞*_*)* can then be presented as follows:

$${\Delta B}_{RGA,n}=\beta_{RGA,n}∙B_{CM,n}$$

$$\Delta B_{\text{RGA},n}=\left[\frac{\frac{{\left(1+i\right)}^{b}-{\left(1+i\right)}^{-n\left(b-r\right)+r}}{{\left(1+i\right)}^{b-r}-1}}{\frac{{\left(1+i\right)}^{b}-{\left(1+i\right)}^{-nb}}{{\left(1+i\right)}^{b}-1}}-1\right]\left[\frac{{\left(1+i\right)}^{b}-{\left(1+i\right)}^{-nb}}{{\left(1+i\right)}^{b}-1}\right]B_{\text{CM}}$$ (19)

$$\Delta B_{\text{RGA},\infty }=\beta_{\text{RGA},\infty }\cdot B_{\text{CM},\infty }=\left[\frac{{\left(1+i\right)}^{b}-1}{{\left(1+i\right)}^{b-r}-1}-1\right]\left[\frac{{\left(1+i\right)}^{b}}{{\left(1+i\right)}^{b}-1}\right]B_{\text{CM}}$$ (20)

## Results & discussion

In the previous section, a theoretical model for the discounted benefits from time savings was developed and an exact multiplicator was derived. In this section, we present (i) an illustration using some numerical estimates of the benefits of using RGA from a meta-analysis, (ii) discuss the advantages and disadvantages of our method, and (iii) conduct a sensitivity analysis. All data underlying this analysis can be consulted in S1 Dataset.

### Benefit analysis

#### Validation of theoretical multiplicator

We start by looking at two studies on wheat breeding by Brennan [10] and Brennan and Martin [11] as a validation of our methodology as these studies report benefit analyses for shortening the breeding process. Since the reported benefits are only due to a shortening of the breeding process, application of our multiplier is also meaningful for wheat. Indeed, several methods exist to speed up the breeding process for wheat [16]. Shortening of the breeding process leads to earlier benefits for both wheat and rice. Brennan [10] estimates the discounted returns from a new wheat cultivar release to be US$ 3,816 × 10^3^; releasing the cultivar one year earlier results in US$ 4,007 × 10^3^ of benefits (5% discount rate). Brennan and Martin [11] asses the incremental value of reducing the breeding process by two years: their base program generates US$ 135.2 × 10^6^ in benefits while their sped-up program leads to US$ 149.0 × 10^6^ (5% discount rate). To compute the benefits from the reduced breeding processes, we apply our proposed multiplicator (1.0500 and 1,1025, respectively) to the base program’s benefits, obtaining US$ 4,006.8 × 10^3^ and US$ 149.058 × 10^6^, respectively). When comparing these results to those presented in the two papers (US$ 4,007 × 10^3^ and US$ 149.0 × 10^6^, respectively), we see their results follow the patterns predicted by the multiplicator in Eq (7)—after rounding. This adds some credibility to our proposed multiplicator method for analysing the benefits from a shortening of the breeding process.

#### Meta-analysis

To measure the potential impact of using RGA, a meta-analysis was performed on recent rice breeding impact studies. Note that our list of included studies (total = 10) is not systematic nor exhaustive since we did not aim at providing a full overview of all rice breeding impact assessments. This overview consists of articles, carefully inspected by the authors, all published after the year 2000 and focusing specifically on the impact of rice breeding.

In order to estimate the potential incremental benefits of adopting RGA, the multiplicator from Eq (7) is applied to the benefits reported in the selected literature for a two-year reduction and the results are shown in Table 3. If multiple discount rates were available, the discount rates in the range 3 to 5% were reported, following Alston et al. [17] who state that “real risk-free rates of interest are typically in the range of from 2 to 5% (p. 24).

**Table 3:** Meta-analysis of incremental societal benefits attributed to various research organisations (Δ*B*_RGA_) for a discount rate (*i*) ranging from 3 to 5% and a two-year reduction (*r*) of the breeding process due to the adoption of RGA in rice breeding (in million US$).

Year published	First author	Region	Crop/technology	Discount rate	Benefit conventional method (reported)	Incremental benefit RGA (imputed)
2002	Mamaril	Philippines and Vietnam	Bt Rice	0.05	618.80	63.43
2004	Pardey	Brazil	Upland rice	0.04	289.18^a^^,^^d^	23.60^a^^,^^d^
2004	Zimmermann	Philippines	Golden Rice	0.03	51.75^a^^,^^b^	3.15^a^^,^^b^
2005	Fan	India	Rice	0.05	30,323.97	3,108.21
		China	Rice	0.05	69,491.02	7,122.83
2005	Singh	Australia	Rice	0.05	50.55	5.18
			Cold-tolerant rice	0.05	91.82	9.41
2006	Stein	India	Golden Rice	0.03	5,087.50^a^^,^^c^	309.83^a^^,^^c^
2007	Jaroensat-hapornkul	Thailand	Rice	0.05	454.86^e^	46.62^e^
2009	Gautam	Eastern India	Rice	0.05	0.41	0.04
				0.05	0.89	0.09
				0.05	0.90	0.09
2011	Brennan	Philippines	Rice	0.05	3,001.40	307.64
		Indonesia	Rice	0.05	7,894.51	809.19
		Vietnam	Rice	0.05	11,129.16	1,140.74
2015	Raitzer	Philippines	Rice	0.05	859.31	88.08
		Bangladesh	Rice	0.05	2,132.43	218.57
		Indonesia	Rice	0.05	10,655.30	1,092.17

*Notes*: Results of some of these studies have been recalculated compared to the original results to allow better integration into our meta-analysis.

^(a)^ Average of optimistic and pessimistic scenario was taken.

^(b)^ Value of US$ 1,030 per DALY was taken.

^(c)^ Value of US$ 1,000 per DALY was taken.

^(d)^ Compounded result.

^(e)^ An exchange rate of 25.135 Thai Baht per US$ was assumed.

Some of the results of these studies have been recalculated compared to the originally reported results to allow better integration into our meta-analysis. The study by Fan et al. [18] reports aggregated benefits and resembles more a breeding program than a single breeding project. Unfortunately, no data are available to make a clear distinction between single breeding projects and breeding programs. For that reason, all incremental benefits are calculated for the conservative scenario where research benefits only come from a single breeding project. Since we showed that a breeding program yields significantly more benefits than a single breeding project, our results provide a conservative estimate of benefits rather than an exact prediction. Although the study by Fan et al. [18] did not use a discount rate, results were still calculated by choosing a discount rate of 5%. The results by Pardey [19] were calculated using compounding rather than discounting. This method converts a benefit stream to a point in time after the beginning of the stream of benefits, using weighing factors that are directly related to the discount rate. However, the formulas presented earlier apply as well in the case of compounding. Our methodology also compounds partially the earlier benefits for studies that did not report any lag in the benefit stream.

Studies on a smaller geographical area such as Gautam [20] (eastern India), Singh et al. [21] (Australia), and Zimmermann and Qaim [22] (Philippines) generate a relatively modest gain when shifting from the conventional pedigree method to RGA. However, based on studies covering larger geographical regions such as Brennan and Malabayabas [23] (Philippines, Indonesia, Vietnam), Fan et al. [18] (India, China), Raitzer et al. [24] (Philippines, Indonesia, Bangladesh), Jaroensathapornkul [25] (Thailand), Mamaril [26] (Philippines, Vietnam), Pardey [19] (Brazil), and the supplementary discussion (online) by Stein et al. [27] (India), we predict a substantial research benefit. Especially in larger countries such as China and India, adopting RGA could generate substantial returns on investment. Using the study undertaken by Fan et al. [18], we even predict a (combined) net research benefit of US$ 10 billion, exceeding IRRI’s 2014 budget by a factor of 100. The studies by Fan et al. [18], Brennan and Malabayabas [23], and Raitzer et al. [24], focusing on the benefits for IRRI in Southeast Asia over a period of from 20 to 25 years, provide a thorough impact assessment and are an eye-opener for the potential of RGA.

### Advantages and disadvantages of our multiplicator

#### Advantages

The advantages of this method for our analysis are threefold. First, it relies on only a single assumption, i.e., benefits are the same for the RGA and conventional breeding method. There is also no need to recalculate the entire benefit model for a second time. Once the original study for the conventional breeding method is conducted, only the discounted benefits and the discount rate are needed. This makes the calculations straightforward and computationally tractable.

Second, a benefit analysis based on this formula is robust to various breeding project or program specifications. Since our multiplier effect is independent of the length of the breeding process, the useful lifespan of the variety and the lag in adoption, the proposed method is readily applicable to almost all rice breeding projects and programs. Whether the method is undertaken for long breeding projects or short projects producing only a few generations causes no bias in calculating the relative increase in benefits. This is especially important as many studies face great difficulty in determining the length of the lifespan of the varieties under study. It is even more challenging to measure the lags in adoption after the breeding process is completed. Some economists have even claimed research lags to be essentially infinite [28]. This great array of lifespans and research lags across different studies can easily be dealt with.

Third, this method does not require the use of a counterfactual as the conventional method acts as the base level to which the incremental benefits are compared. Since these studies are already carried out—often *ex post*—there is much less uncertainty about what would have happened. In fact, this analysis neither looks at what happened, nor what could happen, but at what would have happened if the new technology, RGA in this case, had been used by the rice breeders. This reduces uncertainty and makes benefit estimates more accurate.

#### Disadvantages

The downside to using this method is that it only generates relative changes in benefits. Without numerical estimates of the benefits, no clear picture can be sketched about the potential gains. Internal rates of return and benefit-cost ratios alone—which are often the only economic measures reported in studies—are less useful in evaluating the benefit of reducing the breeding process since the effect of costs cannot be subtracted from the net benefits. It must also be stated that the relative method described here relies heavily on the study under consideration. The incremental benefits calculated here have to be interpreted within the context of the model used by each of the studies, including all its assumptions and uncertainties. Although the methodology is easily applicable to all kinds of breeding projects, over- or underestimations of research benefits will be reflected in the incremental gain from using RGA.

Also, our multiplicator is derived under the assumption that lifespans of varieties remain constant over time. Although RGA is not expected to increase the rate of genetic gain in the short run, the increased intensity of variety release might increase genetic efficiency and possibly also lower the useful lifespan of varieties in the long run. Furthermore, the assumption of constant lifespans may not hold true for some varieties that are developed conventionally. Given decreasing variety lifespans, expected future benefits might be overstated. This limits the applicability of our multiplicator.

### Sensitivity analysis of research benefits and incremental benefits

In the previous section, research benefits were taken as given, i.e., incremental benefits were calculated by multiplying the research benefits with a certain factor. Here, we look at what variables affect research benefits and thus incremental benefits using partial derivatives.

Due to the specific form of the discounting formula used, discounted research benefits are expected to decrease in an exponential way following the discount rate, meaning a rapid decay for low values of the discount rate and a much slower one for higher values. The pattern of research benefits from a single breeding project as a function of the discount rate can be reasonably well approximated through an exponential function of the following form:

$$B_{CM}=\sum_{t=b+m+1}^{b+m+l}\frac{B_{t}}{{\left(1+i\right)}^{t}}\approx K{∙e}^{-ai}$$ (21)

where parameters *a* and *K* are both strictly positive numbers. This curve follows a clear exponential decay from *K* to 0. Parameter *K* represents the value of the undiscounted research benefits, and is thus a measure of the scale of the project under study. Parameter *a* represents the slope of the exponential decay; a higher value of *a* results in a steeper curve. This parameter is a measure of the sensitivity of research benefits to the discount rate. More specifically, parameter *a* refers to the relative lateness of the benefits throughout the lifespan of the variety and thus depends on the time profile of the conventional benefits.

Research and incremental benefits for a two-year reduction were calculated for a range of different discount rates using a spreadsheet-approach for the studies by Singh et al [21], Fan et al. [18], Gautam [20], Brennan and Malabayabas [23], and Raitzer et al. [24]. The study by Raitzer et al. [24] was included twice in this analysis. Firstly, total discounted research benefits were included in Table 3. Second, a specific set of benefits (i.e., DALYs or Disability Adjusted Life Years, saved through reduced hunger) was included in the sensitivity analysis since these data allowed for a more convenient recalculation fitted to our needs. The latter is referred to as Raitzer* et al. [24]. To enable comparison between different studies, the ratio of (discounted) research benefits (*B*_*CM*_) to undiscounted research benefits was taken for every value of the discount rate (relative benefits). Table 4 reports the estimated parameters and R^2^ obtained by OLS regression on the log-transformation of the exponential model [Eq (21)]. As can be seen, this exponential relationship fits the data with an R^2^ of around 99% (Table 4).

**Table 4:** Characteristics of estimated research and incremental benefit functions.

Year publ.	First author	Region	Crop/tech.	*K*	*a*	R^2^	Max. point	Infl. point
2005	Singh	Australia	Rice	80.705	18.52	0.9927	0.05	0.11
			Cold-tolerant rice	81.868	17.38	0.9931	0.06	0.12
2005	Fan	India	Rice	95.146	3.95	0.9915	0.25	0.51
		China	Rice	86.752	6.17	0.9745	0.16	0.32
2009	Gautam	Eastern India	Rice	88.471	11.00	0.9933	0.09	0.18
				87.503	15.08	0.9957	0.07	0.13
				90.477	14.03	0.9971	0.07	0.14
2011	Brennan	Philippines	Rice	76.952	9.23	0.9618	0.11	0.22
		Indonesia	Rice	79.801	14.41	0.9860	0.07	0.14
		Vietnam	Rice	81.873	12.80	0.9867	0.08	0.16
2015	Raitzer*	Bangladesh	Rice	91.645	9.71	0.9955	0.10	0.21
		Indonesia	Rice	88.406	12.90	0.9945	0.08	0.16
		Philippines	Rice	87.273	11.41	0.9919	0.09	0.18

*Note*: Results obtained by OLS regression on the log-transformation of the exponential model [Eq (21)]. To enable comparison between different studies, the ratio of (discounted) research benefits (*B*_*CM*_) to undiscounted research benefits was taken for every value of the discount rate (relative benefits). The maximum and inflexion point for the incremental benefits correspond with $i^{*}=\frac{1}{a}\;$ and $i^{**}=\frac{2}{a}$ respectively.

* The study by Raitzer et al. [24] was included twice in this analysis. Here, a specific set of benefits (i.e., DALYs or Disability Adjusted Life Years, saved through reduced hunger) was included in the sensitivity analysis.

#### Breeding projects

In line with Eq (6), we can make the following approximation for small values of *r*:

$${\beta_{RGA}=(1+i)}^{r}-1\approx ri.$$ (22)

Using the above approximation and Eq (21), the incremental benefits for a reduction of *r* years [see Eq (7)], can be written as:

$${\Delta B}_{RGA}=riK∙e^{-ai}$$ (23)

with partial derivatives:

$$\frac{\partial {\Delta B}_{RGA}}{\partial i}=rK{∙e}^{-ai}(1-ai)$$ (24)

$$\frac{\partial^{2}\Delta B_{\text{RGA}}}{\partial i^{2}}=rKa\cdot e^{-ai}\left(ai-2\right)$$ (25)

$$\frac{\partial {\Delta B}_{RGA}}{\partial K}=ri∙e^{-ai\;}>\;0$$ (26)

$$\frac{\partial {\Delta B}_{RGA}}{\partial a}=-rKi^{2}∙e^{-ai\;}<0$$ (27)

$$\frac{\partial {\Delta B}_{RGA}}{\partial r}=Ki∙e^{-ai}>0.$$ (28)

Eq (24) implies that the incremental benefits (Δ*B*_RGA_) as a function of the discount rate (*i*) show an inverted U-shaped trend. These (theoretical) upward- and downward-sloping patterns can be explained by considering the trade-off between the relative increase in benefits and the benefits from the conventional project. It can be easily seen from Eq (5) that the relative increase in benefits is proportional to the discount rate. For a two-year reduction, the relative increase in benefits rises approximately in a linear way with the discount rate [Eq (6)]. The relative benefit increases as the discount rate does since postponing the benefits will make a bigger difference. However, for each investment, the principle holds that research benefits decrease as the discount rate is raised—this follows from the observation that discounting attaches more weight to early cash flows than to later ones.

Initially, raising the discount rate will increase incremental benefits as postponing the benefits makes a bigger difference but the effect of discounting on the total conventional benefits is still relatively modest. Later on, the effect of the discount rate reduces the value of conventional benefits to the extent that even the effect of bringing benefits earlier cannot compensate completely for the loss: incremental benefits will start to drop. When this moment occurs depends on the sensitivity of benefits coming from the conventional method to the discount rate. Eq (24) states that incremental benefits will reach a peak (maximum) for $i^{*}=\frac{1}{a}$. This point depends on the parameter *a*, and thus on the steepness of the curve. In other words, projects or programs that are more sensitive to the discount rate and thus display more steepness, will reach their maximum sooner. After the peak is reached, incremental benefits per unit of discount rate will drop at an increasing pace until the inflexion point is reached, at $i^{**}=\frac{2}{a}\;$ [Eq (25)]. From this point onwards, incremental benefits continue to drop with the discount rate, but at a decreasing rate. The maximum and the inflexion point for a two-year reduction are reported in Table 4.

**Fig 5:**
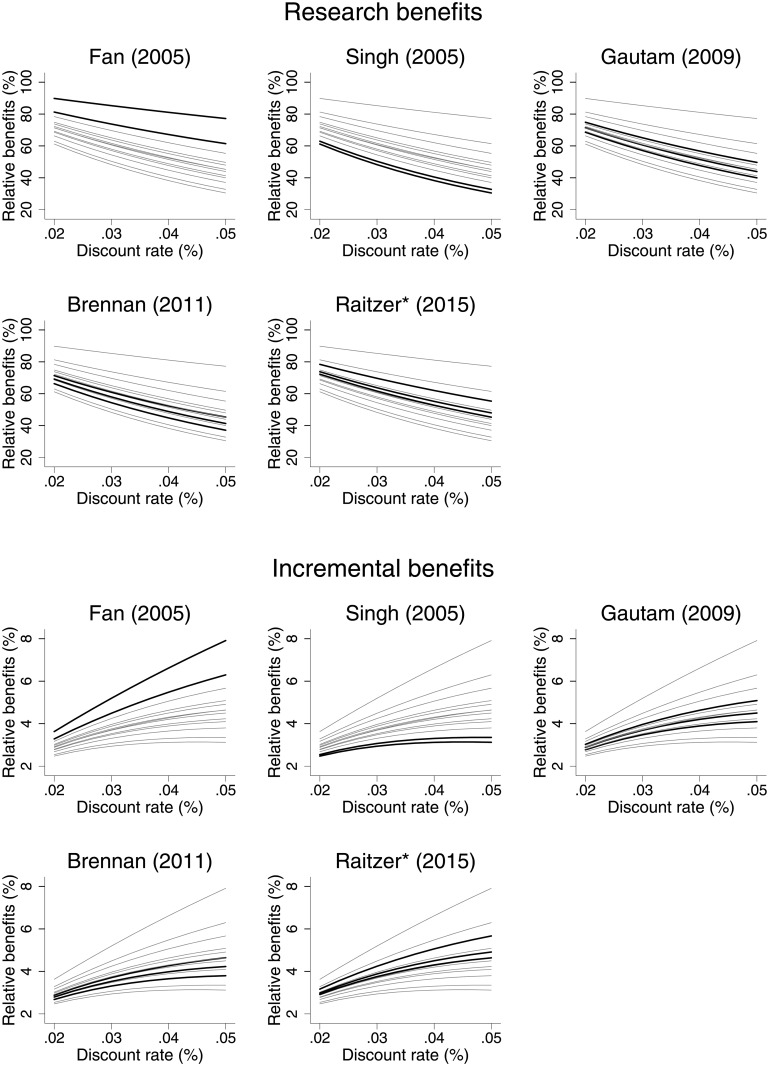
Line plot of relative research benefits for conventional breeding method (upper panel) and incremental benefits (lower panel) for a two-year reduction (*r*) for a meaningful range of discount rates (*i*) for selected breeding studies. *Note*: To enable comparison between different studies, the ratio of (discounted) research (*B*_*CM*_) and incremental benefits (*B*_*RGA*_) to undiscounted research benefits were taken for every value of the discount rate (relative benefits). Different lines represent different breeding projects reported within the same study.

Fig 5 shows a line plot of research benefits (upper panel) and incremental benefits (lower panel) over a meaningful range of discount rates (i.e., from 2 to 5% following Alston et al. [17]) for the selected studies as calculated by our spreadsheet-approach. Again, to enable comparisons between all studies, ratios of discounted research benefits and discounted incremental benefits to undiscounted research benefits are taken. When we compare the upper and lower panels of Fig 5, research benefits are inversely related to the discount rate in contrast to incremental benefits. This observation for the research benefits is not surprising as the approximated exponential model [Eq (21)] predicts a downward-sloping trend over the entire domain. Eq (24), however, predicts that theoretically incremental benefits first rise to a peak and start to drop later as the discount rate proceeds. The downward-sloping part of this trend may lie outside the meaningful range of discount rate. Indeed, Table 4 shows that, for all studies considered, the maximum point is ≥ 5%. Thus, for all meaningful values of the discount rate, incremental benefits will increase following the discount rate. This is an interesting and perhaps counter-intuitive finding as the discount rate is traditionally understood as lowering the current value of future benefit streams.

Studies on research benefits from a single project tend to report a lag in benefits and/or display benefits as a relatively slowly increasing stream in time, potentially followed by a steady state at the maximum. This makes the highest benefits come relatively late in the variety’s lifespan leading to greater sensitivity of research benefit to the discount rate (large parameter *a*). However, studies on programs with a continuous stream of benefits coming from different breeding projects often do not report a lag in benefits and/or display benefits as a relatively constant stream in time. This means benefits come relatively early in the variety’s lifespan (small parameter *a*). As a consequence, the maximum point is much larger than for the simple single project. As noted earlier, the conservative scenario for breeding programs states that research benefits only come from a single breeding project. Fan et al. [18] provide a clear example of this type of study and we can see from Fig 5 (lower panel) that incremental benefits are less robust here compared to other studies. This is in line with the relatively high maximum point as reported in Table 4. Generally, benefit streams with a larger maximum point (small parameter *a*) will have a longer upward sloping trend and are thus less robust to the discount rate in the range of from 2 to 5%.

Next, incremental benefits from shifting to RGA are also smaller—apart from being more robust—for studies that are more sensitive to the discount rate (large parameter *a*), than a comparable study that is less sensitive. This comes from the fact that projects and programs will give lower summed benefits for every value of the discount rate once their sensitivity to it rises. As a consequence, incremental benefits will also be lower for every value of the discount rate. This can be seen clearly from Eq (27): for every value of *i*, incremental benefits drop as sensitivity to discount rate increases (i.e. *a* increases). Thus, when benefits come relatively late in the variety’s lifespan, smaller incremental benefits from RGA are expected.

Also, incremental benefits will always increase as the scale of the project (represented by parameter *K*) is increased, as can be seen from Eq 26. This is intuitively understood as benefits from using RGA are a fixed portion—given the discount rate and reduction in time—of the total research benefits. This is a formal way of saying that larger breeding projects offer more scope of yielding large absolute benefits, as was mentioned earlier.

Lastly, as could be noticed without the sensitivity analysis, reducing the breeding length by more years, results in more benefits (Eq 28). In other words, when more years can be saved, more benefits will be achieved. This is obvious as earlier benefits will always be worth more due to the process of discounting. This effect is analogous for reducing the lag in adoption.

#### Breeding programs

Similar to Eq (22), we can make use of the general approximation that (1 + *i*)^b^ − 1 ≈ *bi*. This approximation is less precise than the one in Eq (6) because higher order effects are disregarded when b > 2, but since we only want to know the direction of the different factors affecting benefits for breeding programs, this approximation is still acceptable. In the same line of reasoning, we perform our sensitivity analysis only for the infinite-projects scenario. Using the above approximation, the incremental benefits from an infinite number of breeding projects for a reduction of *r* years [Eq (20)] can be rewritten as:

$${{\Delta B}_{RGA,\infty }=B}_{CM}\frac{\left(1+bi\right)}{bi}[\frac{b}{\left(b-r\right)}-1]$$ (29a)

$${{\Delta B}_{RGA,\infty }=B}_{CM}\frac{r(1+bi)}{b(b-r)i}$$ (29b)

$${\Delta B}_{RGA,\infty }=Ke^{-ai}∙\frac{r(1+bi)}{b(b-r)i}$$ (29c)

with partial derivatives:

$$\frac{\partial {\Delta B}_{RGA,\infty }}{\partial i}=\frac{r(abi^{2}+ai+1)}{b(r-b)i^{2}}{K∙e}^{-ai}<0$$ (30)

$$\frac{\partial {\Delta B}_{RGA,\infty }}{\partial a}=\frac{r(bi+1)}{b(r-b)}{K∙e}^{-ai}<0$$ (31)

$$\frac{\partial {\Delta B}_{RGA,\infty }}{\partial K}=\frac{r(bi+1)}{b(b-r)i}{∙e}^{-ai}>0$$ (32)

$$\frac{\partial \Delta B_{\text{RGA},\infty }}{\partial b}=-\frac{r\left(b^{2}i+2b-r\right)}{b^{2}{\left(b-r\right)}^{2}i}K\cdot e^{-ai}<0$$ (33)

$$\frac{\partial \Delta B_{\text{RGA},\infty }}{\partial r}=\frac{\left(bi+1\right)}{{\left(r-b\right)}^{2}i}K\cdot e^{-ai}>0$$ (34)

Unlike with breeding projects, the discount rate has an unambiguous relation to incremental benefits from an infinite breeding program: with a rising discount rate, incremental benefits will always decrease [Eq (30)]. This comes from the observation that raising the discount rate makes future breeding projects less valuable and thus decreases total benefits, outweighing the effect of more valuable earlier benefits. For the meaningful range of discount rates (from 2 to 5%), breeding processes (from 10 to 20 years), time savings (from 1 to 3 years) and parameter *a* (from 10 to 20, see Table 4), the following empirical relation holds: $i^{*}=\frac{1}{a+0.45nb}$. Using the meaningful range of parameters, we can easily calculate that programs with at least 10 breeding projects (i.e., 9 repetitions of the inception breeding project, or *n* = 9) approximate the limit to infinity well enough to display the same inverse relation to the discount rate. For a smaller number of breeding projects, incremental benefits may still theoretically show both a rising and declining trend to the discount rate for given values of the parameters.

Eqs (31) and (32), respectively, show that incremental benefits (Δ*B*_RGA_) will always decrease with parameter *a* and will always increase with parameter *K*. Eq (34) states that when more years can be saved, more benefits will be achieved. This is in line with the findings for breeding projects.

Here, we also look at the effect of the breeding process. From Eq (33), it is clear that for shorter breeding processes, more incremental benefits can be achieved using RGA. This can be explained as follows. Consider that two breeding programs have the same reduction in time, but a different breeding process. Each new breeding project within the program with the longer process will start later than the equivalent breeding project from the other breeding program. As a consequence, each project will be worth less for the program with the longer process due to discounting. The parameter is not present in Eq (5), for single breeding projects. This is because a stream of benefits will be worth relatively more if it starts earlier, but this relative increase is independent on when the research benefits started. However, when different breeding projects are added cumulatively, this difference starts to matter.

## Conclusions

In the past, rice breeding policy has been a key instrument in the developing world, especially in Southeast and South Asia and Africa. Recently, interest is growing in transforming conventional rice breeding methods, especially with the aim of accelerating the breeding process. Although acceleration of breeding processes has been recognised as a profitable investment in the past, not much is known about the scale and underlying dynamics of the associated incremental benefits. To date, evidence of the profitability of accelerating the plant breeding process has been case-specific only and generally lacking for rice breeding altogether.

To estimate the incremental benefits generated by accelerated breeding processes, a novel and straightforward methodology was theoretically developed. A great advantage of our multiplicator is that it is applicable to all benefit models, irrespective of the benefit pattern. This is crucially important as benefit patterns in the literature and in reality follow a wide variety of functional forms. Our approach not only foregoes the need of specifying a single benefit pattern over time, but is also independent of the length of the variety’s lifespan. Additionally, the approach taken is straightforward and can be implemented as a multiplicator effect for the much-used but often unwieldy benefit structure models. Moreover, these benefit structure models do not need to be known once the discounted research benefits are given. As a rule of thumb, the incremental benefits from a two-year reduction in time equal 10% of research benefits at the 5% discount level.

To extend the current base of literature of the plant breeding process acceleration, this method was applied to a meta-analysis of recent rice breeding impact assessments. After analysing a list of studies, shortening of breeding processes times appears to be a worthwhile investment. For varieties with a large geographical spread and for large breeding programs, benefits can add up to several billion US dollars. This figure highlights evidently the enormous potential of reducing breeding processes, even for small reductions of one or two years. These findings also reveal the tremendous responsibility that lies with breeders and policy makers in the public rice breeding sector. Ideally, breeders should weigh the substantial benefits foregone of using an additional year for breeding against any potential benefits of their conventional breeding method. Future research might include a larger set of impact assessments, not necessarily restricted to rice, to provide more empirical evidence for our proposed methodology.

This methodology was completed with a carefully worked-out sensitivity analysis to create a better understanding of the incremental benefits generated by accelerated breeding processes. An important finding is that shortening of plant breeding not only generates benefits but these benefits are even greater for early peaking adoption programs (i.e., maximum adoption level reached early in the variety’s lifespan). As a policy implication, this stresses the need for carefully selecting which varieties might benefit most from an accelerated breeding process. Varieties that are expected to be adopted rapidly form the preferred choice of applying RGA to. Perhaps counterintuitively, this study provides evidence that incremental benefits from accelerated breeding processes are not inversely related to the discount rate for all meaningful ranges of that discount rate. This can be an essential insight for policy makers at IRRI when comparing impact assessment with different discount rates. As a hands-on instruction, policy makers should be sceptical of relatively high discount rates, potentially inflating the incremental benefits for reduced timing upwards.

This study potentially has some limitations. First, we assumed that the lifespan of the variety remains the same and annual benefits are comparable irrespective of the breeding method. We then extended this assumption to multiple breeding projects (breeding program). Although RGA is not expected to increase the rate of genetic gain in the short run, the increased intensity of variety release might increase genetic efficiency and possibly also lower the useful lifespan of varieties in the long run. Future research might extend our methodology by allowing for lifespans that decrease over time. Second, our analysis relies on approximated functional forms. Future studies might experiment with different functional forms and add more parameters of interest.

## Supporting information

S1 Dataset Selected rice benefit studies (data). (XLSX)
